# Mast Cells and Nerve Signal Conduction in Acupuncture

**DOI:** 10.1155/2018/3524279

**Published:** 2018-03-07

**Authors:** Na Yin, Hongwei Yang, Wei Yao, Ying Xia, Guanghong Ding

**Affiliations:** Shanghai Key Laboratory of Acupuncture Mechanism and Acupoint Function, Department of Aeronautics and Astronautics, Fudan University, Shanghai 200433, China

## Abstract

Nerve and mast cells are densely distributed around acupoints in connective tissue. To explore the internal relations between them in acupuncture effect, we examined dorsal root potential (DRP) response to acupuncture at Zusanli (ST36) under sodium cromoglicate (DSCG, a mast cell stabilizer) intervention in anesthetized Sprague-Dawley (SD) rats. We used single unit nerve recording techniques to collect nerve signals from DRP afferent nerves for a 45-minute period that includes 4 stages, that is, base, drug absorption, acupuncture, and recovery stages. We analyzed the recorded signals from time-domain and frequency-domain perspectives. The results showed that once acupuncture needle was inserted, twisting needle excited more nerves discharges than those at base discharges in ACU (from 35.1 ± 7.2 to 47 ± 9.2 Hz, *P* = 0.004), and there existed the same trend in Saline + ACU group (from 23.8 ± 2.6 to 29.8 ± 4.2 Hz, *P* = 0.059). There was no change of nerve discharges under twisting needle with injection of DSCG (from 34.8 ± 5.3 to 34.7 ± 4.4 Hz, *P* = 0.480). We conclude that acupuncture manipulation promotes neural signal production and DSCG could partly inhibit nerve discharges.

## 1. Introduction

Acupuncture has been used for analgesia, treating visceral function disorders, and improving motor functions [1–3]. As a mechanical stimulation, acupuncture activates nerve impulse and then generates action potentials, leading neural signal transmission from peripheral to central nervous system. Acupuncture stimulation can induce the inhibition of mechanical hypersensitivity induced by spinal nerve ligation [4] and improve the cardiovascular response [5]. A destruction (e.g., blocking the peripheral nerve or the nerve pathway, or partial damaging the central nervous system) can largely inhibit acupuncture analgesia effect [6, 7]. So the primary mechanism whereby acupuncture elicits the body response appears to be through the activation of afferent nerve fibers innervating the skin and muscles by acupuncture needle stimulation [3]. Histological studies confirmed that mast cells are densely distributed around acupoints of rats and along the small blood vessels and nerve capsule [8, 9]. Sodium cromoglicate (DSCG), a mast cell stabilizer, can suppress mast cell activation and degranulation via inhibiting calcium ion influx [10]. Some studies showed that sciatic nerve discharges were attenuated and acupuncture analgesic effect was reduced by DSCG intervention, suggesting that mast cells at acupoints participate in the priming process of nerve signals [8, 11]. However, sciatic nerve is a compound nerve and its nerve signals are bidirectional; therefore it is difficult to explain exactly the activation mechanism of nerve signals.

In this study, we recorded nerve signal discharges with different acupuncture manipulations under DSCG pretreatment. Our experiments suggest that mast cells affected nerve signal conduction and acupoints activation induced by acupuncture.

## 2. Materials and Methods

### 2.1. Animals

The experiments were performed in accordance with guidelines of the Animal Care and Use Committee of Shanghai Research Center for Acupuncture and Meridians. Male Sprague-Dawley (SD) rats (180–230 g) were purchased from Shanghai Lab, Animal Research Center, and housed in cages with a temperature-controlled environmental (22–25°C) and a 12/12-hours light/black cycle. Food and water were made available ad lib. All animals were handled with care to prevent infection and to minimize stress. All behavior experiments were performed between 9 am and 5 pm. For each experimental group, animals were chosen randomly.

### 2.2. Surgery

**Figure 1:**
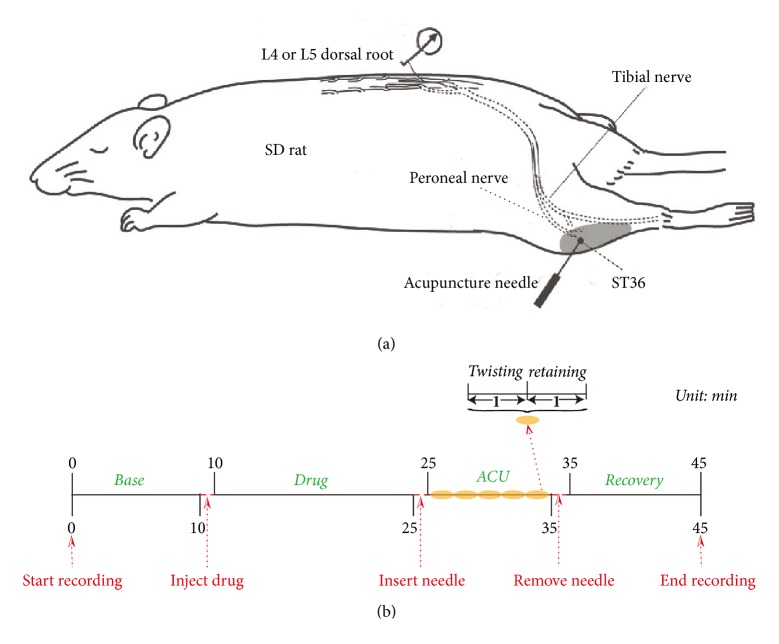
*Schematic diagram of the experimental procedures.* (a) Nerve signals were recorded from SD rat. Acupuncture needle was inserted into ST36 in acupuncture stage modified from Kagitani et al. [36]. (b) The whole experiment was recorded for 45 min including 4 stages, that is, base, drug absorption, acupuncture, and recovery stages.

After anesthetization with 10% chloral hydrate (0.4 ml/100 g i.p), animals' rectal temperature was maintained at 37.5 + 0.5°C by a thermostatically regulated heating pad (ATB 1100, Nihon Kohden, Tokyo). A dorsal laminectomy was performed between the spinal levels of T13-L5. After opening the capsule of spinal cord with artificial cerebrospinal fluid (ASCF) (in mM: NaCl 130, KCl 3.5, NaHCO_3_ 24, NaH_2_PO_4_ 1.25, MgCl_2_ 1.2, Glucose 10, CaCl_2_ 1.2), the dorsal root of the L4 or L5 spinal nerves was separated and cut close to the spinal cord. These nerves were covered with warm liquid paraffin and the peripheral cut segments were placed on unipolar platinum-iridium wire recording electrodes. The temperature of the liquid paraffin bath was maintained at 37.5 + 0.2°C to avoid any effects of temperature. Dissection of the dorsal roots was performed under microscope (Leica, M520) until we recorded single unit nerve activity. A single afferent fiber was identified by the identical shape of the discharge spikes. Single unitary potentials were amplified (DAGAN, EX4-400, Japan 300–3000 Hz, Gain 500 set) to transmit to computer via multichannel recorder (powerlab 16/30, ADInstruments, Australia) and were visually collected and displayed on software (version 7.3, ADInstruments, Australia) (Figure 1(a)).

### 2.3. Acupuncture Stimulation

After shaving the hair of the hindlimb, a stainless steel acupuncture needle (0.35 mm in diameter, 25 mm in length, Suzhou Kangnian Medical Devices Co., Ltd., Suzhou) was inserted into the skin and underling muscles. The stimulation point was within an area of approximately 2 cm^2^ around rat's ST36 (located approximately 5 mm lateral and distal to the anterior tubercle of the tibia). The perpendicular needling depth was approximately 7 mm. We alternately twisted right and left with fingers once every second for 1 min and retained needle without any manipulation for 1 min, which lasted 5 cycles for 10 min.

### 2.4. Nerve Signal Recording and Animal Grouping

The experiments included four stages: base stage (Base) for 10 min, drug absorption stage (Drug) for 15 min, acupuncture stage (ACU, including twisting needle once every second for 1 min and then retaining needle for 1 min, 5 cycles) for 10 min, and recovery stage (Recovery) for 10 min (Figure 1(b)). SD rats were randomly divided into 3 groups. The ACU group (*n* = 9) was treated only with acupuncture manipulation in acupuncture stage without any operation including drug injection; 20 *μ*L normal Saline was injected to ST36 before drug absorption stage in Saline + ACU group (*n* = 12). Twenty *μ*L [8] DSCG (0.02 g/mL in normal Saline vehicle, DSCG from Sigma-Aldrich) was injected to ST36 before drug absorption stage in DSCG + ACU group (*n* = 8).

### 2.5. Data Analysis

The quality of neural signal recording is defined by their signal-to-noise (S/N) ratios [12]. In most cases, only electrodes with S/N ratios larger than three were recorded so that single units could be extracted. In this study, a single afferent fiber was identified by the identical shape of the discharge spikes. Experimental data was exported by Labchart recording and analyzed by MATLAB. To effectively analyze results and reduce individual differences influence, we selected nerve discharges of base stage as the standard value and calculated the nerve discharges difference value between other stages and base stage in each group for analyzing the differences among groups. Statistical significance of differences of each value between groups was analyzed by independent sample *T*-test and correlation of relative factors of groups by paired *T*-test.

## 3. Results

### 3.1. DRP Responses to Different Operations and Different Drug Interventions

**Figure 2:**
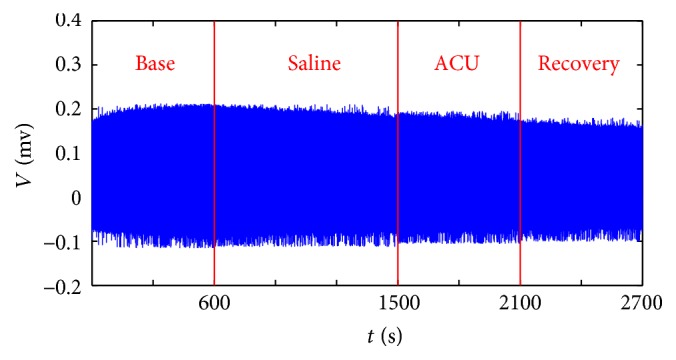
*Representative recordings of nerve discharges of Saline + ACU group.* The experiments included four stages: base stage (Base) for 10 min, drug absorption stage (Drug) for 15 min, acupuncture stage (ACU, including twisting needle once every second for 1 min and then retaining needle for 1 min, 5 cycles) for 10 min, and recovery stage (Recovery) for 10 min.

**Figure 3:**
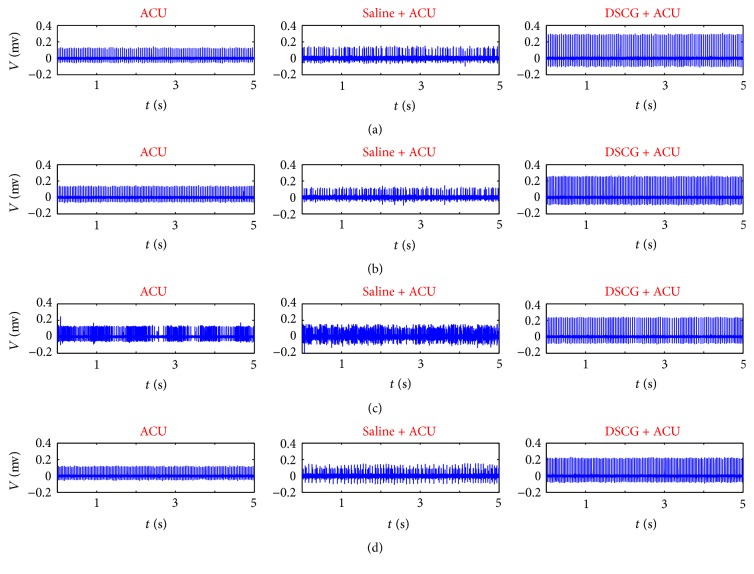
*5-second recordings of nerve discharges at each stage of ACU, Saline + ACU and DSCG + ACU.* From top to bottom, (a) is base stage, (b) is drug absorption stage, (c) is acupuncture stage, and (d) is recovery stage.

**Figure 4:**
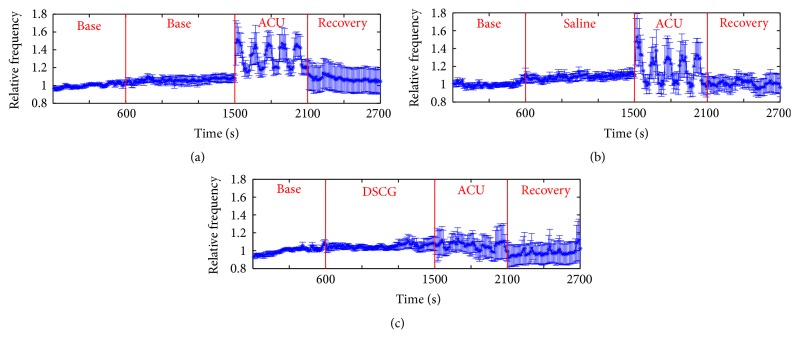
*Time course (2700s) of nerve discharges under different operations and different drug pretreatments.* (a) Without drug injection on 9 animals (ACU group). (b) With Saline injection on 12 animals (Saline + ACU group). (c) With DSCG injection on 8 animals (DSCG + ACU group). Ordinate is the average of 10 s of all animals in one group comparing to base mean and “1” represents the mean value of nerve discharges during base stage; the vertical lines indicate the SEM. *P* = 0.018 (a), *P* = 0.008 (b), and *P* = 0.036 (c) when comparing drug mean versus base mean, *P* = 0.004 (a), *P* = 0.059 (b), and *P* = 0.480 (c) when comparing twisting mean versus base mean, *P* = 0.017 (a), *P* = 0.386 (b), and *P* = 0.484 (c) when comparing retaining mean versus base mean, and *P* = 0.109 (a), *P* = 0.340 (b), and *P* = 0.210 (c) when comparing recovery mean versus base mean in respective group ((a), (b), and (c), paired *T*-test).

In order to investigate real-time nerve discharges, we recorded nerve signals throughout the experiment for 45 min (Figures 2–4). Nerve discharges after different drugs injection rose from 23.8 ± 2.6 to 25.4 ± 2.6 Hz in Saline + ACU group (*P* = 0.008) and from 34.8 ± 5.3 to 36.1 ± 5.4 Hz in DSCG + ACU group (*P* = 0.036), which was consistent with changes of nerve discharges in ACU group (from 35.1 ± 7.2 to 38.1 ± 7.8 Hz, *P* = 0.018). However, there were no differences in the drug-induced discharges among these three groups (ACU, 3.0 ± 1.2 Hz; Saline + ACU, 1.6 ± 0.6 Hz; DSCG + ACU, 1.2 ± 0.5 Hz; *P* > 0.1). It revealed that nerve discharges had no direct correlation with drug pretreatments. After acupuncture needle was inserted, twisting needle excited more nerves discharges than those at base discharges in ACU (from 35.1 ± 7.2 to 47 ± 9.2 Hz, *P* = 0.004), and there existed the same trend in Saline + ACU group though there was no statistical significance yet (from 23.8 ± 2.6 to 29.8 ± 4.2 Hz, *P* = 0.059). There was no change in nerve discharges under twisting needle with injection of DSCG (from 34.8 ± 5.3 to 34.7 ± 4.4 Hz, *P* = 0.480). Moreover, there was no significant difference in the increased discharges induced by twisting needles between ACU and Saline + ACU group (11.9 ± 3.3 Hz in ACU group versus 5.9 ± 3.5 Hz in Saline + ACU group, *P* = 0.118). However, DSCG injection (0.3 ± 1.8 Hz) induced a stronger inhibition (versus 11.9 ± 3.3 Hz in ACU group, *P* = 0.005). It showed that acupuncture manipulation played an important role in acupuncture effect while injection of DSCG could damage acupuncture effect. Once acupuncture needle was removed, nerve discharges all recovered to base level (ACU, from 39.9 ± 9.7 to 35.1 ± 7.2 Hz, *P* > 0.1; Saline + ACU, from 24.5 ± 3.5 to 23.8 ± 2.6 Hz, *P* > 0.1; DSCG + ACU, from 31.7 ± 4.7 to 34.8 ± 5.3 Hz, *P* > 0.1), and there were no statistical differences in the increased discharges during recovery stage among the three groups (ACU, 4.7 ± 3.5 Hz; Saline + ACU, 0.6 ± 1.5 Hz; DSCG + ACU, −2.6 ± 2.8 Hz *P* > 0.05). These results suggest that the acupuncture aftereffect might be mainly through none nerve mechanism.

### 3.2. Effect of Acupuncture Manipulation

**Figure 5:**
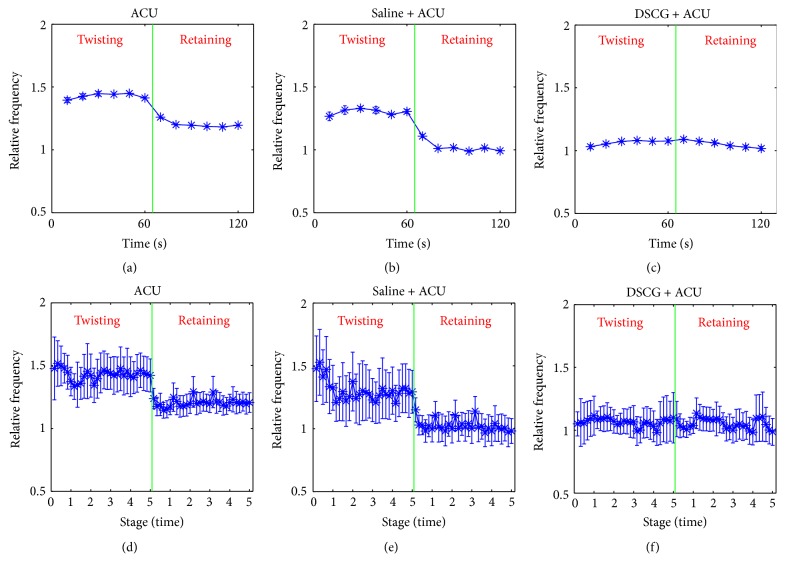
*Nerve discharges throughout acupuncture stage.* (a) Nerve discharges of all twisting and retaining stages versus base discharges mean in ACU group. (b) Nerve discharges of all twisting and retaining stages versus base discharges mean in Saline + ACU group. (c) Nerve discharges of all twisting and retaining stages versus base discharges mean in DSCG + ACU group. (d) Nerve discharges of 5 independent twisting and retaining stages versus base discharges mean in ACU group. (e) Nerve discharges of 5 independent twisting and retaining stages versus base discharges mean in Saline + ACU group. (f) Nerve discharges of 5 independent twisting and retaining stages versus base discharges mean in DSCG + ACU group. *P* = 0.002* (a), P* = 0.025* (b), *and *P* = 0.461* (c)* when comparing the twisting-retaining discharges ratio in respective group ((a), (b), and (c), paired *T*-test). *P* = 0.227, *P* = 0.020, and *P* = 0.036 when comparing the twisting-retaining discharges ratio in the whole acupuncture stage ((a) versus (b), (a) versus (c), and (b) versus (c), independent sample *T*-test), *P* < 0.05 when comparing the twisting-retaining discharges ratio in any one of five independent stage ((d) versus (e), (d) versus (f), and (e) versus (f), independent sample *T*-test). Ordinate is the average of 10 s of all animals in one group comparing to the mean at base stage with “1” representing the mean value of nerve discharges in base stage (vertical lines indicate the SEM).

To analyze the impact of acupuncture manipulation on curative effect, twisting and retaining needles were used alternately after acupuncture needle was inserted into ST36 (Figure 5). In contrast to retaining needle, twisting needle increased much more discharges in ACU group (from 42.0 ± 9.0 Hz to 47.0 ± 9.2 Hz, *P* = 0.002) and in Saline + ACU group (from 24.5 ± 3.6 to 29.8 ± 4.2 Hz, *P* = 0.025), but there was no difference between twisting and retaining needle in DSCG group (from 34.7 ± 4.4 to 34.6 ± 4.8 Hz, *P* = 0.461). In the discharge ratio of twisting needle to retaining needle, there was no significant difference between ACU and Saline + ACU groups (1.2 ± 1.3 in ACU versus 1.3 ± 0.1 in Saline + ACU, *P* = 0.227). However, the injection of DSCG (0.9 ± 0.1) showed a stronger inhibition on nerve excitement (*P* = 0.020, versus ACU; *P* = 0.036, versus Saline + ACU). Therefore, twisting needle had advantages in inducing nerve discharges over retaining needle, which existed in the five independent acupuncture stages among the ACU and Saline + ACU groups (Figures 5(d)-5(e), Table 1).

### 3.3. Characteristics of Nerve Discharges in Interspike Interval

**Figure 6:**
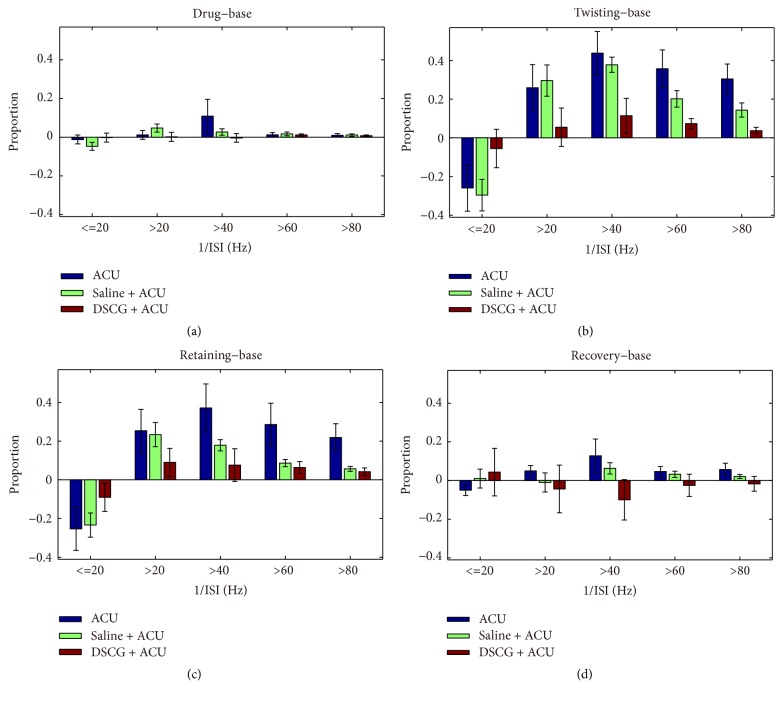
*Nerve discharges distribution in interspike interval.* (a) The added value of 1/ISI proportion when comparing drug versus base stage. (b) The added value of 1/ISI proportion when comparing twisting versus base stage. (c) The added value of 1/ISI proportion when comparing retaining versus base stage. (d) The added value of 1/ISI proportion when comparing recovery versus base stage. The following listed *P* values were calculated in 1/ISI over 40 Hz. (a) *P* = 0.286, *P* = 0.124, and *P* = 0.118, the value-added of 1/ISI proportion when comparing drug mean versus base mean, (b) *P* = 0.390, *P* = 0.050, and *P* = 0.010, the value-added of 1/ISI proportion when comparing twisting mean versus base mean, (c) *P* = 0.459, *P* = 0.048, and *P* = 0.009, the value-added of 1/ISI proportion when comparing retaining mean versus base mean, and (d) *P* = 0.204, *P* = 0.181, and *P* = 0.099, the value-added of 1/ISI proportion when comparing recovery mean versus base mean (ACU versus Saline + ACU group, ACU versus DSCG + ACU group, and Saline + ACU versus DSCG + ACU group, independent sample *T*-test).

To analyze the characteristics of nerve discharges, we defined the reciprocal of interspike interval (1/ISI) as the instantaneous frequency and selected 1/ISI of base stage as the standard. We calculated the proportion differences of other stages minus base stage in each group (Figure 6). The nerve discharges after drug absorption stage were similar to base discharges in frequency (1/ISI) over 40 Hz among the three groups (ACU, from 40% to 50%, *P* > 0.1; Saline + ACU, from 20% to 20%, *P* > 0.1; DSCG + ACU, from 50% to 50%, *P* > 0.1; Figure 6(a)). Meanwhile, there were no differences among the three groups (*P* > 0.1). In contrast to base discharges, once acupuncture needle was inserted into ST36, nerve discharges of twisting needle increased by 40% in ACU (from 40% to 80%, *P* = 0.003) and Saline + ACU group (from 20% to 80%, *P* < 0.01) in frequency (1/ISI) over 40 Hz. Besides, nerve discharges of retaining needle increased by 30% compared to those of base stage both in ACU (from 40% to 70%, *P* = 0.008) and Saline + ACU group (from 20% to 50%, *P* < 0.01) in frequency (1/ISI) over 40 Hz. However, in DSCG + ACU group, there was no major difference of nerve discharges under acupuncture stage—including twisting (from 50% to 60%, *P* = 0.06) and retaining needle (from 50% to 60%, *P* = 0.14)—comparing to base discharges in frequency (1/ISI) over 40 Hz (Figures 6(b)-6(c), *P* < 0.05, versus ACU; *P* < 0.05, versus Saline + ACU). In recovery stage, we also did not observe any difference as comparing to base discharges in ACU (from 40% to 50%, *P* > 0.1) and Saline + ACU (from 50% to 40%, *P* > 0.1) in frequency (1/ISI) over 40 Hz. Unexpectedly, there existed a minor difference of nerve discharges between recovery and base stages in Saline + ACU group (from 20% to 30%, *P* = 0.036) in frequency (1/ISI) over 40 Hz. The data suggest that acupuncture changed nerve discharges density in frequency (1/ISI) over 40 Hz and injection of DSCG inhibited nerve discharges.

## 4. Discussion and Conclusions

This study is aimed at exploring the interaction between mast cells and nerve signals in acupuncture effect using single unit nerve recording techniques from DRP afferent nerves in the model of anesthetized SD rats. Our results showed that acupuncture manipulation induced more nerve discharges and the acupuncture-induced nerve discharges could be inhibited by a mast cell stabilizer, suggesting that acupuncture effects are dependent, at least partially, on the role of the mast cells.

### 4.1. The Selection of Nerve Signals Recording Method

It is well known that many recording techniques are applied to peripheral nerve signals collection, such as electrode arrays inserted in the peripheral nerve [13, 14], cuff electrodes wrapped in nerve for compound action potentials [15], and intrafascicular microelectrode implanted in the nerve [16]. However, the above recording techniques are very complicated and unstable. What is more, they almost collect bidirectional signals for analysis. In this study, we used single unit nerve recording technique [16] in anesthetized rats to record specific afferent nerve fibers in the dorsal roots at the 4th or 5th lumbar segments. Recording from the dorsal spinal root eliminated the recording of antidromically activated efferent nerves, which specifically defined the groups of afferent fibers activated. Thus, we inserted the acupuncture needle around ST36 area underling skin and muscles and twisted it manually to record activities of single nerve fibers from a dissected nerve branch of the 4th or 5th lumbar spinal dorsal roots.

### 4.2. The Influence of Acupuncture Manipulation in Acupuncture Effect

The present study showed that acupuncture manipulation had a close relation with nerve signal conduction (Figures 2–6). Traditionally, the reaction of acupuncture manipulation, known as “de qi” sensation, is the one of the most important components in acupuncture therapeutic effect, including numbness, heaviness, soreness, and distension. Sham acupuncture involving needle insertion without manipulation stimulation at acupoints did not generate acupuncture effect [5]. Some studies confirmed that the needling sensation caused by lifting-thrusting was stronger than that of twisting-rotating manipulation [17]. Nerve network is a complex system. The complexity of DRP of lifting-thrusting was even higher than twisting-rotating manipulation through complexity analysis method to study [18]. These data suggest that there exist significant differences in nerve signals response to different acupuncture manipulations. Meanwhile acupuncture sensation was also related to the depth of acupuncture needle [19]. What is more, studies suggested that the manipulation of lift-thrusting and rotation mechanical stimulus caused local connective tissue deforming, which induced the cellular level signals potentially spreading along connective tissue via mechanotransduction [20]. Once the structure of collagen fibers at ST36 was destroyed by injection of type I collagenase, the analgesic effects of rotation or lift-thrusting manipulation were also attenuated [21]. The above studies suggest that acupuncture manipulation played an important role in acupuncture curative effect.

### 4.3. The Exploration of the Acupuncture Aftereffect

We found that the acupuncture aftereffect might not be generated through nerve signal conduction (Figures 4 and 6). The acupuncture aftereffect refers to the lasting effect after the cease of acupuncture. In the clinical study of acupuncture treating migraine, the therapeutic effect at acupoints versus nonacupoints appeared to work after four weeks of the cease of acupuncture [22]. Meanwhile through functional magnetic resonance imaging (fMRI) method, newly increased functional connectivity was found at 25 min after removing acupuncture needle in human brain [23]. However, the acupuncture aftereffect may be related to category and stage of disease, individualized acupuncture reaction, acupuncture methods, intervals between treatments, and therapeutic course [24].

### 4.4. The Role of Mast Cells in Acupuncture Effect

In the present study, we confirmed that nerve discharges due to acupuncture (including twisting and retaining needle) were restrained with DSCG injection. Since DSCG is a mast cell stabilizer, we concluded that mast cells participated in acupuncture effect and affected nerve signal conduction. Meanwhile mast cells focused on acupoints and the density was higher than a nearby sham point [8]. Furthermore, mast cells are preferentially colocalized with sensory neurons, partly innervated directly by nerves and degranulated at the site of a nerve lesion [25]. DSCG intervention could largely reduce mast cell degranulation and suppress nerve discharges [8, 11]. So mast cell degranulation induced by acupuncture was closely related to neural signal transmission.

### 4.5. The Interactions between Mast Cells and Nerve Fibers

There were studies showing the activation of different populations of afferent nerve fibers during acupuncture stimulation [26–30]. Based on gate control theory, for example, somatic stimulations such as acupuncture, massage, cupping, and scraping can activate partial A fibers. Afferent inputs of A fibers can block the pain signals carried by C-fibers at the spinal cord level and thus stop the transmission of pain signals to the brain [30]. The peripheral mechanism theory suggests that acupuncture can excite mechanoreceptors in the tissues around the acupoint. Acupuncture stimulation is converted into nerve impulses by these receptors and the impulses are transmitted through fibers II and III [30]. Meanwhile, groups II, III, and IV fibers were activated during manual acupuncture in humans [26], while groups III and IV fibers were activated in rats [5]. Some experiments suggested acupuncture can induce mast cell degranulation [8, 31]. The products of activated mast cells could also overtly stimulate nerve endings, causing long-lasting changes in neuronal excitability [29]. Besides, in the study of neuronal-based systems of allergy, allergen challenge and mast cell mediator released in a sensitized trachea ex vivo does not overtly activate nociceptive A*δ* cough nerves but instead lowers their activation threshold to a mechanical stimulus [28]. In the lower airways, allergic mediators including histamine can increase the excitability of certain C-fibers to the point that the mechanical perturbation of eupneic breathing leads to their activation [27]. However, these studies mostly focused on the activation of nerve fibers by acupuncture stimulation; there was no consistent conclusion on the action mechanism between the somatic afferent fiber types and mast cells.

### 4.6. The Underlying Relationship between Mast Cell Mediators and Acupuncture Effects

Mast cells can release lots of tryptase, histamine, and 5-HT through degranulation after acupuncture stimulation [32]. Histamine increased the pain threshold in adjuvant-induced arthritic rats, producing an analgesic effect though mast cell degranulation blocked by disodium cromolyn, which suggests acupuncture analgesic effects may be histamine-dependent [31]. Other research reported histamine activated C-fibers during “itch” sensation [33]. Histamine released from mast cells after intestinal anaphylaxis stimulates mesenteric afferents via 5-HT_3_ and histamine H_1_ receptors [34]. In the cocultures of superior cervical ganglia (SCG) and rat basophilic leukemia cells (RBLs), ATP released from activated mast cells could affect Ca^2+^ response to activate nerves [35]. Therefore, mast cell mediators may affect nerve discharges, thus participating in acupuncture signal conduction.

In summary, the present study demonstrated that mast cells could affect nerve signal conduction in acupuncture effect.

## Figures and Tables

**Table 1:** Nerve discharge throughout acupuncture stage.

Stage	Nerve discharges frequency ($\overset{-}{x}$ ± SEM, Hz)					
	ACU		Saline + ACU		DSCG + ACU	
	Twist	Retain	Twist	Retain	Twist	Retain
1	47.5 ± 8.8	41.1 ± 8.5	32.4 ± 4.6	24.8 ± 3.4	35.3 ± 5.2	35.1 ± 5.2
2	45.6 ± 8.9	41.7 ± 8.8	28.7 ± 4.0	24.5 ± 3.8	36.4 ± 5.1	36.7 ± 5.3
3	47.8 ± 10	43.0 ± 9.7	28.7 ± 4.3	24.6 ± 3.6	35.4 ± 4.9	34.6 ± 5.1
4	47.1 ± 9.2	42.2 ± 9.0	29.2 ± 4.4	24.5 ± 3.8	33.5 ± 4.2	33.3 ± 4.8
5	47.0 ± 9.1	42.1 ± 9.0	29.7 ± 4.3	23.9 ± 3.7	33.0 ± 3.9	33.2 ± 4.4
